# The Human Milk Protein-Lipid Complex HAMLET Sensitizes Bacterial Pathogens to Traditional Antimicrobial Agents

**DOI:** 10.1371/journal.pone.0043514

**Published:** 2012-08-15

**Authors:** Laura R. Marks, Emily A. Clementi, Anders P. Hakansson

**Affiliations:** 1 Department of Microbiology and Immunology, University at Buffalo, State University of New York, Buffalo, New York, United States of America; 2 The Witebsky Center for Microbial Pathogenesis and Immunology, University at Buffalo, State University of New York, Buffalo, New York, United States of America; 3 New York State Center of Excellence in Bioinformatics and Life Sciences, Buffalo, New York, United States of America; Colorado State University, United States of America

## Abstract

The fight against antibiotic resistance is one of the most significant challenges to public health of our time. The inevitable development of resistance following the introduction of novel antibiotics has led to an urgent need for the development of new antibacterial drugs with new mechanisms of action that are not susceptible to existing resistance mechanisms. One such compound is HAMLET, a natural complex from human milk that kills *Streptococcus pneumoniae* (the pneumococcus) using a mechanism different from common antibiotics and is immune to resistance-development. In this study we show that sublethal concentrations of HAMLET potentiate the effect of common antibiotics (penicillins, macrolides, and aminoglycosides) against pneumococci. Using MIC assays and short-time killing assays we dramatically reduced the concentrations of antibiotics needed to kill pneumococci, especially for antibiotic-resistant strains that in the presence of HAMLET fell into the clinically sensitive range. Using a biofilm model in vitro and nasopharyngeal colonization in vivo, a combination of HAMLET and antibiotics completely eradicated both biofilms and colonization in mice of both antibiotic-sensitive and resistant strains, something each agent alone was unable to do. HAMLET-potentiation of antibiotics was partially due to increased accessibility of antibiotics to the bacteria, but relied more on calcium import and kinase activation, the same activation pathway HAMLET uses when killing pneumococci by itself. Finally, the sensitizing effect was not confined to species sensitive to HAMLET. The HAMLET-resistant respiratory species *Acinetobacter baumanii* and *Moraxella catarrhalis* were all sensitized to various classes of antibiotics in the presence of HAMLET, activating the same mechanism as in pneumococci. Combined these results suggest the presence of a conserved HAMLET-activated pathway that circumvents antibiotic resistance in bacteria. The ability to activate this pathway may extend the lifetime of the current treatment arsenal.

## Introduction

*Streptococcus pneumoniae* (the pneumococcus) is a major cause of respiratory tract infections including otitis media, sinusitis, sepsis, meningitis and pneumonia and is associated with significant morbidity and mortality worldwide [1]. Although three safe approaches to anti-pneumococcal vaccination are available, protection with these vaccines is limited [2]–[5]. Therefore, the pneumococcus remains a major driver of healthcare utilization even in developed nations, with more than 4 million illnesses causing more than 22,000 deaths in the US alone [5]. In developing nations the situation is markedly worse; pneumonia represents the single leading cause of death in children under five, with *S. pneumoniae* being the main bacterial etiology, alone being responsible for about 11% of all deaths in children in this age group [6], [7].

The clinical situation is further complicated by the emergence of antibiotic resistance. Since the discovery of penicillin, 17 different classes of antibiotics have been produced [8]. Antibiotic use has become widespread and a cornerstone of medical treatment – being used to treat infections ranging from the seriously life-threatening to the more trivial and frequently non-bacterial illnesses. This constant antibiotic pressure, combined with the ability of pneumococci to incorporate DNA from other strains and closely related species, has led to the evolution and acquisition of resistance traits [9]–[11]. Multiple-antibiotic-resistant strains are now widespread and bacteria have developed at least one mechanism of resistance (and frequently many more) to every single antibiotic class [8], [12]–[14].

This rise in single- and multi-drug resistant bacteria has limited the number of agents that produce reliable treatment for these infections, spurring efforts to develop alternative antimicrobial therapies. Leading candidates are metabolites, peptides and enzymes produced by organisms that kill bacteria under natural conditions [15], [16].

Human milk has evolved to promote both infant health and wellbeing [17]. Epidemiologic studies indicate that breastfeeding decreases the incidence and severity of a range of infectious diseases including bacterial meningitis, bacteremia, diarrhea, respiratory tract infection, and otitis media [7], [18]–[21]. Protection against infection is provided by an array of protective factors, including innate immune factors, secretory antibodies, lipids, carbohydrates, oligosaccharides, lysozyme, and lactoferrin, as well as components that we are most certainly not yet aware of [17], [22].

Our studies investigating the direct-acting antimicrobial factors present in human milk identified a novel antimicrobial complex HAMLET (Human α-lactalbumin made lethal to tumor cells) [23], [24]. HAMLET was purified from the casein fraction of human milk, and found to consist of alpha-lactalbumin in a partially unfolded conformation that is stabilized under physiological conditions by the human milk-specific fatty acids oleic and linoleic acid [25], [26]. This protein-lipid complex has a potent, yet specific bactericidal effect against the respiratory tract pathogens *Streptococcus pneumoniae*, with some activity also against *Haemophilus influenzae* and some strains of *Moraxella catarrhalis* in vitro, whereas other species of bacteria are resistant [24].

In this study we characterized the ability of HAMLET to potentiate the effect of the antibiotics gentamicin, erythromycin and penicillin against both sensitive and resistant pneumococcal strains. We showed a significantly increased activity of combination therapy on pneumococcal biofilms both in vitro and in a mouse model of nasopharyngeal colonization. The potentiating effect of HAMLET was so strong that antibiotic-resistant strains grown in biofilms or colonizing the murine nasopharynx could be effectively eradicated in the presence of HAMLET at concentrations effective against sensitive strains. The mechanism of this potentiation required a calcium influx into the bacteria as well as kinase activation, similar to the mechanism required for HAMLET-induced death of pneumococci. Finally, HAMLET's antibiotic-sensitizing effect is not confined to species that HAMLET alone can kill, but is also present in species against which HAMLET alone has no activity, suggesting that many bacterial species possess similar death mechanisms that can be explored to develop novel anti-bacterial agents.

## Results

### HAMLET lowers the minimal inhibitory concentration of penicillin, erythromycin, and gentamicin, especially in resistant strains

We have earlier shown that pneumococci resistant to a several classes of antibiotics are equally sensitive to HAMLET death, suggesting that HAMLET uses a different mechanism of action than these agents [24]. This was confirmed in this study as all strains included in the study, whether sensitive or resistant to antibiotics, had the same minimal inhibitory concentration (MIC) for HAMLET. Based on the increasing use of combination therapy in infectious diseases, we were therefore interested in investigating the potential synergistic effects between HAMLET and common antibiotics.

**Figure 1:**
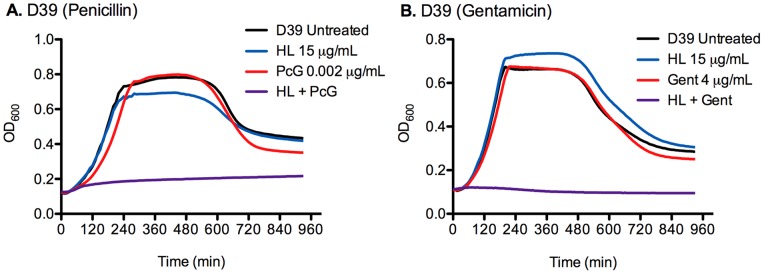
HAMLET lowers the MICs of gentamicin, erythromycin and penicillin. *S. pneumoniae* D39 were grown in broth for 16 hours in the presence of penicillin G (A) or gentamicin (B) with and without the addition of HAMLET. The figure shows representative growth curves for the lowest concentration of antibiotic and HAMLET that inhibited bacterial growth by combination treatment without either agent alone affecting growth.

In the absence of HAMLET, the MIC of the penicillin-compound penicillin G was 0.01 μg/ml for both the penicillin-sensitive strains *S. pneumoniae* strains D39 (serotype 2) and EF3030 (serotype 19F, from a child with otitis media) (**Table 1**). Simultaneous presence of subinhibitory concentrations of HAMLET (15 µg/ml for this batch or 0.75X its MIC) reduced the MIC of penicillin G five-fold, to 0.002 μg/ml (**Figure 1A**). To investigate whether the potentiation of HAMLET held true also in pneumococcal strains resistant to penicillin G, the penicillin-resistant serotype 6A otitis media strain SP670 with an MIC of 4 μg/ml was tested in a similar way. In the presence of 0.75X MIC of HAMLET, this strain became susceptible to penicillin showing a 20-fold decreased MIC of 0.2 μg/ml, that was significantly more decreased than the penicillin-sensitive strains (*P*<0.05; **Table 1**). Importantly, HAMLET-potentiation had the ability to place this strain in the penicillin-sensitive range, where penicillin would again be a potentially useful therapeutic agent.

**Table 1:** MIC determination of HAMLET/antibiotic combination therapy.

Bacterial Strain	MIC (µg/mL)			Fold reduction
	HAMLET	Penicillin	Penicillin + HL	
***S. pneumoniae*** ** D39**	20	0.01	0.002*	5
***S. pneumoniae*** ** EF3030**	20	0.01	0.002*	5
***S. pneumoniae*** ** SP670**	20	4	0.2*	20
***A. baumanii*** ** AB307**	>1,000	>100	25***	>4
***A. baumanii*** ** AB979**	>1,000	>100	25***	>4
***M. catarrhalis*** ** 7169**	>1,000	>50	1.56***	>32
***M. catarrhalis*** ** BC8**	750	>50	12.5***	>4

	HAMLET	Erythromycin	Erythromycin + HL	
***S. pneumoniae*** ** D39**	20	0.03	0.01*	3
***S. pneumoniae*** ** JY53**	20	3	0.01*	300

	HAMLET	Gentamicin	Gentamicin + HL	
***S. pneumoniae*** ** D39**	20	16	4*	4*
***S. pneumoniae*** ** D39**	20	16	2**	8**
***S. pneumoniae*** ** EF3030**	20	16	4*	4*
***S. pneumoniae*** ** EF3030**	20	16	2**	8**
***A. baumanii*** ** AB307**	>1,000	36	4.5***	8
***A. baumanii*** ** AB979**	>1,000	2.5	1.25***	2
***M. catarrhalis*** ** 7169**	>1,000	25	6.7***	4
***M. catarrhalis*** ** BC8**	750	25	6.7***	4

* HL (HAMLET) used at a concentration of 0.75X MIC (15 µg/mL).

** HL (HAMLET) used at a concentration of 0.85X MIC (17 µg/mL).

*** HAMLET used at a concentration of 50 µg/mL.

The same pattern was true for the macrolide erythromycin. The addition of 0.75X MIC of HAMLET, reduced the MIC of erythromycin for the sensitive strain D39 3-fold and the MIC of the erythromycin-resistant strain JY53, (carrying an erythromycin resistance cassette in the *pspA* locus) a dramatic 300-fold (*P*<0.001) to 0.01 µg/ml, making this strain highly susceptible to this antibiotic and equally sensitive as the non-resistant D39 strain in the presence of HAMLET (**Table 1**).

Finally, the MIC of the aminoglycoside gentamicin, to which pneumococci show relative tolerance, was reduced 4-fold in the presence of 0.75X MIC of HAMLET for both *S. pneumoniae* D39 and EF3030 (**Figure 1B** and **Table 1**). Moreover, the addition of 17 μg/ml of HAMLET (85% of the MIC) decreased the MIC of gentamicin for both strains 8-fold, indicating that a sub-inhibitory concentration of HAMLET could sensitize *S. pneumoniae* to gentamicin in a concentration-dependent manner (**Table 1**). Combined these results suggest that HAMLET potentiates the anti-pneumococcal effects of various classes of antibiotics with a significantly better potentiation occurring in antibiotic-resistant strains.

### HAMLET potentiates short-time pneumococcal kill by gentamicin, penicillin and erythromycin

To further verify the potentiation effect of HAMLET for common antibiotics we performed short-time killing assays. Similar to the MIC values, all strains, irrespective of antibiotic sensitivity, were equally sensitive to HAMLET and required a concentration of approximately 15 times the MIC concentration to eradicate each inoculum in 1 hour, a further support that HAMLET uses a different activation mechanism than the antibiotics used in this study.

Gentamicin at high concentrations reduced the inoculum 1.4 log_10_ after 1 hour of incubation, whereas both penicillin G and erythromycin lacked any bactericidal activity alone against the sensitive strains D39 and EF3030, even at concentrations as high as 5,000 µg/ml and 1,000 µg/ml, respectively, over that period of time. Treatment with these antibiotics was therefore performed over 4 hours. For each of these experiments, HAMLET and each antibiotic were titrated to produce less than 1 log_10_ kill in sensitive strains, respectively, over the incubation time, and those concentrations were then used to perform combination treatments.

**Figure 2:**
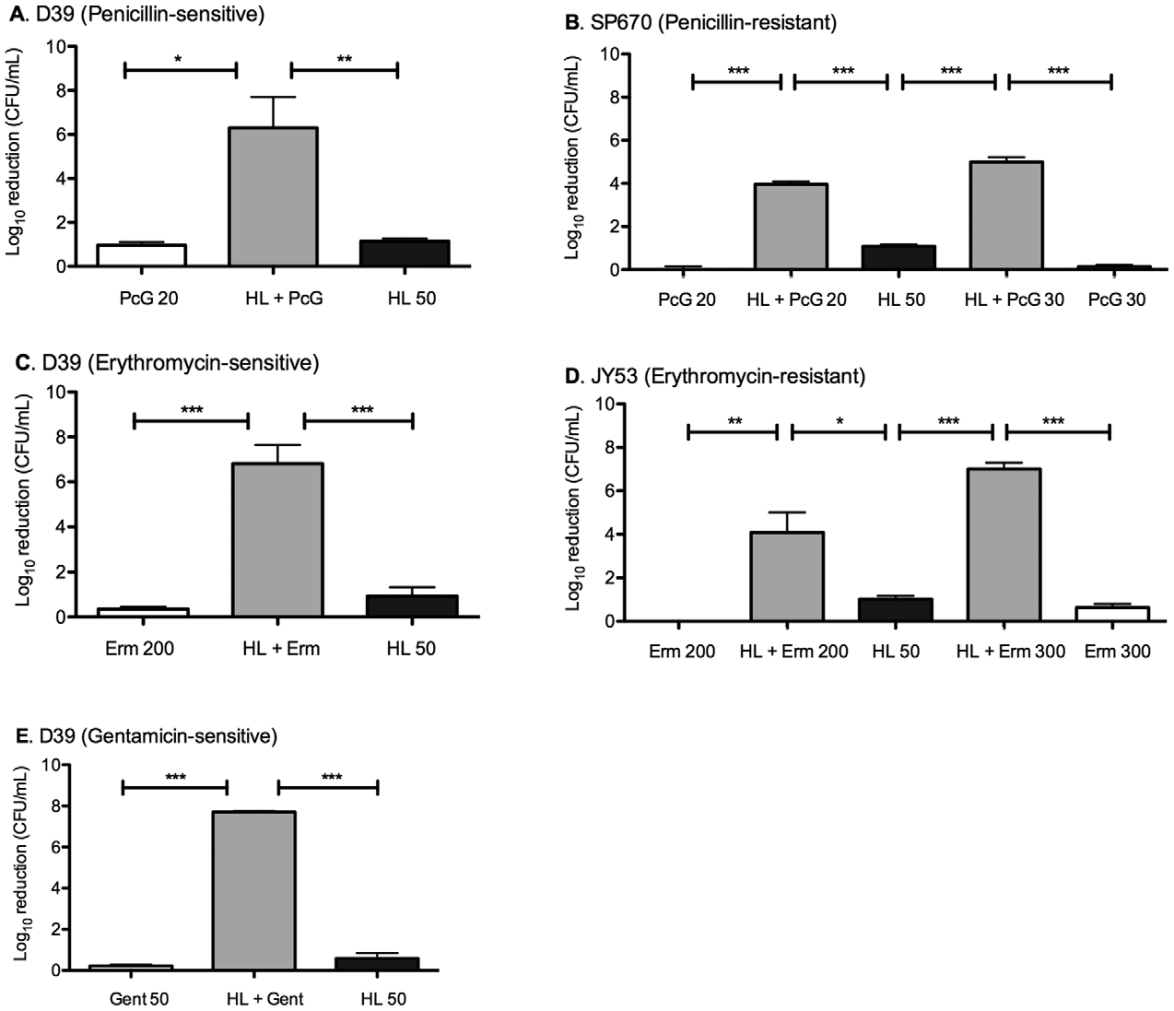
Potentiation of short-time pneumococcal killing by gentamicin, penicillin and erythromycin in the presence of HAMLET. Short-time killing of the penicillin-sensitive strain D39 (A) by penicillin G (20 µg/mL), HAMLET (50 µg/mL) and penicillin combined with HAMLET over 4 hours. (B) Killing of the penicillin-resistant strain SP670 by penicillin G (20 or 30 µg/mL), HAMLET (50 µg/mL), or penicillin G combined with HAMLET over 4 hours. (C) Killing of the erythromycin-sensitive strain D39 by erythromycin (200 µg/mL), HAMLET (50 µg/mL), or erythromycin combined with HAMLET over 4 hours. (D) Killing of the erythromycin-resistant D39-derivative JY53 by erythromycin (200 µg/mL), HAMLET (50 µg/mL), or erythromycin combined with HAMLET over 4 hours. (E) Killing of D39 by gentamicin (50 µg/mL), HAMLET (50 µg/mL), or gentamicin combined with HAMLET over 1 hour. The results are based on three individual experiments with duplicate samples and are expressed as means ± S.D. Statistics was performed using the unpaired Student t-test. Significance was indicated as follows: * = *P*<0.05, ** = *P*<0.01, *** =  *P*<0.001, ns  =  not significant.

For penicillin G, a range of concentrations from 1 to 100 µg were tested, with the most successful combination effect seen using 50 µg/ml of HAMLET and 20 µg/ml of penicillin G that alone caused no bactericidal activity (**Figure 2A**). However, combination treatment of the two agents resulted in significantly higher killing than the added killing of each agent alone (*P*<0.05) with near eradication of the 7.5 log_10_ bacterial inoculum (**Figure 2A**). To obtain a similar activity with HAMLET alone, more that 5-fold higher concentrations were required, and penicillin failed to kill more than 2.6 log_10_ by itself, even at a 50-fold higher concentration (1,000 µg/ml) for 4 hours. The potentiation effect was also present in the penicillin-resistant strain SP670 that had a 20-fold higher MIC than the D39 strain, using the same concentrations of penicillin G and HAMLET (*P*<0.001 compared to the additive killing effect of both agents alone; **Figure 2B**). Increasing the penicillin concentration to 30 µg/mL (that had no bactericidal activity by itself) resulted in even more effective synergistic killing (*P*<0.001; **Figure 2B**).

Similar and even stronger effects were seen when a sublethal concentration of HAMLET was combined with erythromycin. As with penicillin G, a range of erythromycin concentrations (1–1,000 µg/ml) was tested and concentrations that produced no bactericidal activity alone but synergistic effects with HAMLET were used. Combination of 50 µg/ml of HAMLET and 200 µg/ml of erythromycin resulted in significantly increased killing than the added killing of each agent alone after 4 hours (P<0.001) with near-eradication of the inoculum (**Figure 2C**). When using the erythromycin-resistant strain JY53 that displayed a 100-fold higher MIC, combination treatment with the two agents at the same concentrations was somewhat less effective but still caused a bactericidal activity significantly higher than the additive killing of each agent alone (*P*<0.05), which could be increased to complete eradication of the bacterial inoculum when 300 µg/ml erythromycin (*P*<0.001) was used together with 50 µg/mL HAMLET, a highly significant synergy in killing of erythromycin-resistant pneumococci (**Figure 2D**).

Finally, the effect of combination treatment with HAMLET and gentamicin using 50 µg/ml of HAMLET or 50 µg/ml of the aminoglycoside gentamicin induced bactericidal activity significantly enhanced compared with the additive killing of the two agents (*P*<0.001) with the entire bacterial inoculum eradicated after only 1 hour of incubation (**Figure 2E**). This was substantial as gentamicin was unable to kill more than 1.4 log_10_ by itself, even at concentrations up to 1,000 µg/ml.

Combined these results suggest that HAMLET acts as a powerful bactericidal potentiator producing synergistic effects with several classes of antibiotics and is able to significantly decrease the antibiotic concentrations needed to induce death of pneumococci.

### HAMLET/antibiotic combination treatment potentiate killing of in vitro biofilms

To address the role of HAMLET's potentiating effect on antibiotic function under physiological conditions, we first subjected pneumococci growing as biofilms to combination treatment with HAMLET and antibiotics. We and others have shown that pneumococci colonizing or infecting the mucosal surfaces of the host grows primarily in aggregated communities or biofilms that are well known to display substantially increased resistance to antibiotics as well as other antimicrobial agents [27]–[32].

**Figure 3:**
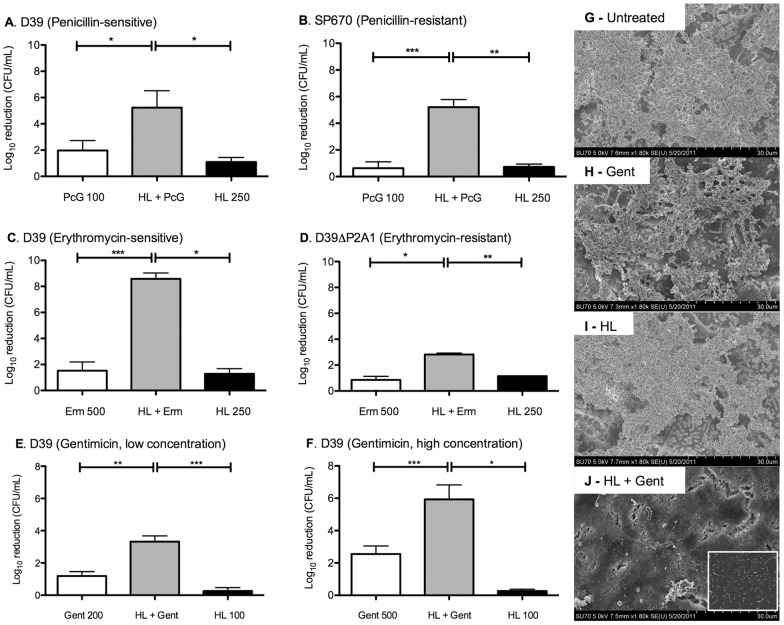
The effect of HAMLET/antibiotic combination treatment on in vitro biofilm viability. The activity of penicillin G (100 µg/mL), HAMLET (250 µg/mL), or the combination of both agents were tested on *in vitro* biofilms of the penicillin-sensitive strain D39 (A) or the penicillin-resistant strain SP670 (B) formed over a prefixed epithelium of NCI-H292 cells and were tested by determining the bacterial death (in log_10_) after culturing bacterial dilutions overnight on blood agar. Similarly, the activity of erythromycin (250 µg/mL), HAMLET (250 µg/mL), or the combination of both agents on *in vitro* biofilms of the erythromycin-sensitive strain D39 (C) or the erythromycin-resistant strain D39-P2A1 (D) were tested in a similar fashion as was the activity of 200 µg/ml gentamicin (E) or 500 µg/ml gentamicin (F) alone or in combination with 100 µg/ml HAMLET (100 µg/mL) over 3 hours on pre-established biofilms formed by the strain EF3030. The results are based on three individual experiments with duplicate samples. Statistics was performed using the paired Student t-test. Significance was indicated as follows: * = *P*<0.05, ** = *P*<0.01. To visualize the morphology of the treated biofilms, SEM studies were performed. Images show (G) the structure of an untreated 48 hour EF3030 biofilm, (H) an EF3030 biofilm after 3 hour treatment with 500 µg/mL gentamicin alone, (I) an EF3030 biofilm after 3 hour treatment with HAMLET (100 µg/mL) alone and (J) an EF3030 biofilm after 3 hour treatment with the combination of 100 µg/mL HAMLET and 500 µg/mL Gentamicin. An epithelial substratum prior to biofilm formation has been included as a control (insert in panel J). The increased bactericidal activity of the combination treatment was associated with a reduction in the density of adherent bacteria and biofilm matrix.

Pneumococcal biofilms were formed with D39 pneumococci for 48 hours over a pre-fixed epithelial substratum. We used an epithelial substratum rather than an abiotic substratum as we have recently shown that biofilm growth on epithelial cell surfaces results in biofilms with higher biomass, higher antimicrobial resistance and more structural resemblance to biofilms observed during nasopharyngeal colonization in vivo than biofilms grown on abiotic surfaces [27]. Various concentrations of HAMLET and penicillin alone or in combination were tested to find the optimal synergy. When treating mature biofilms with 250 µg/ml of HAMLET and 100 µg/ml of penicillin that by themselves were not bactericidal, as defined by at least 3 log_10_ death of the bacteria, the effect was significantly enhanced with 5.2 log_10_ pneumococci killed out of the 8.3 log_10_ total biofilm biomass, which was significantly higher than the additive effect of the two agents (P<0.05; **Figure 3A**).

HAMLET's anti-biofilm potentiation of was even more evident when the penicillin-resistant strain SP670 was used. Mature biofilms formed by this strain over a pre-fixed epithelial substratum showed almost complete resistance to treatment with either HAMLET (250 µg/mL) or penicillin (100 µg/mL) alone. However the combination of HAMLET and penicillin demonstrated a dramatic and synergistic bactericidal effect, significantly higher than the additive effect of the two agents (P<0.01; **Figure 3B**).

Even more pronounced results were seen for erythromycin. D39 biofilms formed for 48 hours and treated with erythromycin alone (500 µg/ml) showed no bactericidal effects (**Figure 3C**). In contrast, the combination of erythromycin with 250 µg/ml HAMLET resulted in a synergistic increase in anti-biofilm activity with the near eradication of the biofilm biomass, which was significantly higher than the additive effect of both agents (*P*<0.05; **Figure 3C**). The effect on the erythromycin-resistant strain JY53 was less pronounced with a more additive effect observed after treatment with 500 µg/mL erythromycin in combination with 250 µg/ml HAMLET, which was not significantly different from the additive effect of each agent (*P* = 0.12; **Figure 3D**).

Finally, pneumococcal biofilms formed with EF3030 pneumococci for 48 hours were treated with 100 μg/mL HAMLET and either 200 µg/ml or 500 μg/mL gentamicin alone or in combination for 3 hours. Neither concentration of gentamicin was bactericidal, whereas the combined treatment of the biofilms with both agents for 3 hours induced a bactericidal activity, which were both significantly higher than the additive effect of both agents (*P*<0.05 and *P*<0.01, respectively for 200 and 500 µg/ml gentamicin; **Figure 3E and F**). Scanning electron microscopy of EF3030 biofilms 3 hours after treatment with a combination of both HAMLET and gentamicin also demonstrated a marked change in the appearance of the biofilm, with near eradication of all adherent bacteria from the epithelial cell substratum (**Figure 3J**). In contrast, treatment with either agent alone yielded only minimal reductions in the density of adherent bacteria and matrix (**Figure 3H and I**) compared to untreated biofilms (**Figure 3G**).

### Increased eradication of nasopharyngeal colonization in mice using combined antibiotic-HAMLET treatment

Pneumococci produce complex biofilms in the nasopharynx during asymptomatic colonization that are highly resistant to antimicrobial treatment [27]. We therefore evaluated the ability of HAMLET to potentiate the activity of traditional antibiotics in a murine colonization model *in vivo*.

**Figure 4:**
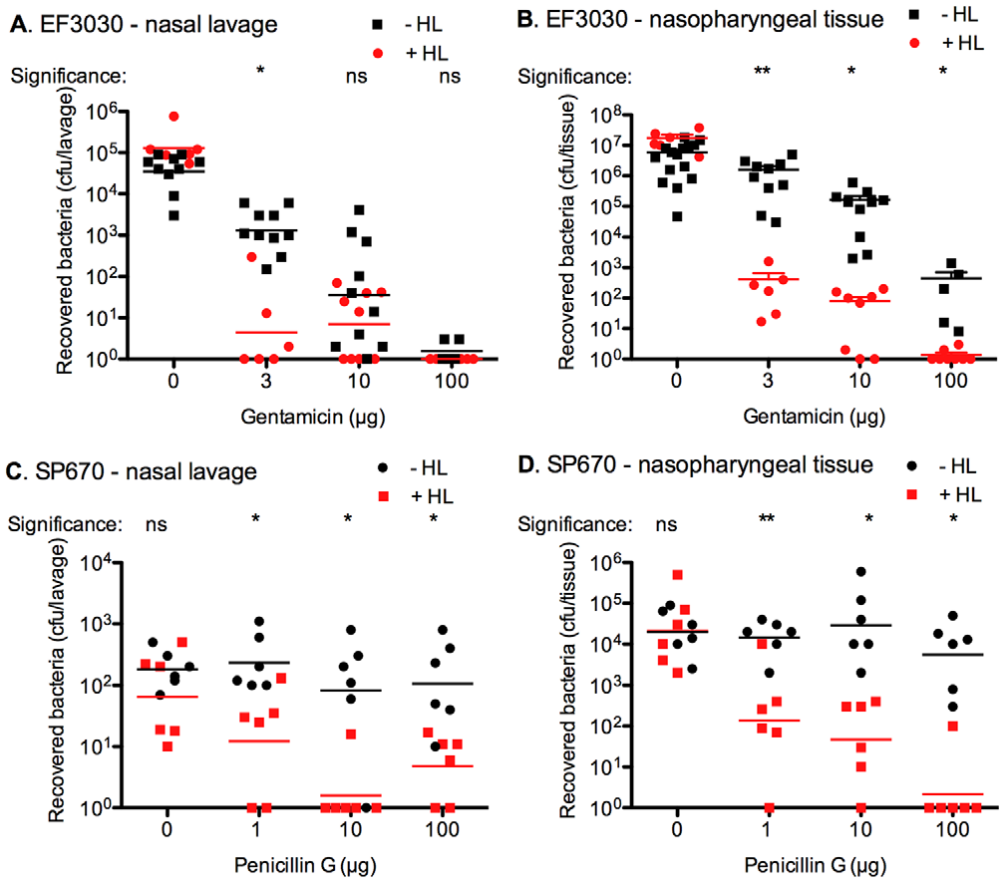
HAMLET-antibiotic combination treatment eradicates pneumococci during nasopharyngeal colonization. (A and B) Mice were colonized with *S. pneumoniae* EF3030 for 48 hours, treated intranasally with various doses of gentamicin in the presence (red circles) or absence (black squares) of HAMLET (50 µg) for 6 hours, and the bacterial burden associated with the nasal lavage (A) and the nasopharyngeal tissue (B) was determined. Bacteria in nasal lavage and associated with the nasopharyngeal tissue were significantly more sensitive to gentamicin/HAMLET combination therapy than gentamicin alone. (C and D) Mice were colonized with the penicillin-resistant strain *S. pneumoniae* SP670 (MIC  = 4 µg/mL) for 48 hours, treated intranasally with various doses of penicillin G in the presence (red circles) or absence (black squares) of HAMLET (50 µg) for 12 hours and the bacterial burden associated with the nasal lavage (C) and the nasopharyngeal tissue (D) was determined. Penicillin G alone had no effect on the bacterial burden in either the nasal lavage or in the tissue. However, combination therapy with HAMLET and penicillin caused a dose-dependent decrease in bacterial burden leading to eradication of colonization. The graph shows colonization data for individual mice, with the mean recovered bacteria and the standard deviation depicted. The results are based on experiments using groups of 6–10 mice. Statistics was performed using the unpaired Student t-test. Significance was indicated as follows: * = *P*<0.05, ** = *P*<0.01, ns  =  non-significant.

Bacteria were inoculated intra-nasally for 48 hours with *S. pneumoniae* EF3030 and the established bacterial colonization was then treated once with increasing doses of gentamicin alone or in combination with 50 μg HAMLET locally in the nares for 6 hours. The viability of bacteria both in a nasal wash and the nasopharyngeal tissue was measured by viable counts. Colonized mice treated with vehicle alone (phosphate-buffered saline) showed a colonization rate of 4×10^6^ CFU per tissue with an approximate 10-fold lower presence of bacteria in the nasal wash (7×10^5^ CFU/ml). Mice treated with HAMLET alone showed no decrease in bacterial burden either in the tissue or in the nasal lavage, whereas gentamicin alone caused a slow reduction of the bacterial burden starting at a dose of 3 µg/mouse in the nasal lavage and 10 µg/mouse in the nasopharyngeal tissues (**Figures 4A and B**).

However, mice treated with a combination of the two agents showed a significantly increased death of the bacteria removed by nasal lavage, compared with those treated with gentamicin alone (**Figure 4A**). A statistically significant reduction of the bacterial burden in the presence of HAMLET was evident already at a dose of 3 µg of gentamicin (150 µg/ml) whereas a 10-fold higher dose (1,500 µg/ml) was required to eradicate nasal lavage-associated bacteria with gentamicin alone. Bacteria associated with the tissue were highly resistant to gentamicin and substantial growth was detected even after a dose of 100 µg (5,000 µg/ml), 10 times the killing dose in the short time killing assay in vitro (**Figure 4B**). However, in the presence of HAMLET, a comparable decrease in tissue associated bacterial load was obtained using a 33-fold lower dose of gentamicin (3 µg; **Figure 4B**).

Using the same procedure, mice we also colonized intra-nasally for 48 hours with the penicillin-resistant strain SP670 and then treated with increasing doses of penicillin alone or in combination with 50 μg HAMLET locally in the nares, and bacterial viability in the nasal lavage and nasopharyngeal tissue was assessed after 12 hours. Untreated mice showed a colonization rate of ∼1×10^5^ CFU associated with each nasopharyngeal tissue with only around 2×10^2^ CFUs present per 100 µl nasal wash. HAMLET treatment alone resulted in no decrease in the pneumococcal burden. In mice treated with increasing does of penicillin alone both tissue-associated bacteria and lavage-associated bacteria were completely resistant to penicillin treatment up to an intranasal dose of 100 µg (5,000 µg/ml) (**Figure 4C and D**). In contrast, in the presence of 50 µg of HAMLET, near complete eradication of all lavage-associated bacteria was observed using 10 µg penicillin (*P*<0.05) and fewer than 10 colony forming units per lavage was detected at 100 µg penicillin, which was not statistically different from the effect of the 10 µg treatment (**Figure 4C**). Similarly, a significant decrease (2 log_10_) in colonization of the nasopharyngeal tissue was seen already at 1 µg penicillin (50 µg/ml), a dose 100-fold lower than in mice treated with penicillin alone over the same treatment period, and at 100 µg/ml all the bacteria were eradicated in the presence of HAMLET with no change in colonization observed with penicillin G alone (**Figure 4D**).

These results support the fact that HAMLET's potentiating effects on antibiotic activity functions under in vivo conditions and can potentiate the effect of antibiotics against strains resistant to the same antibiotic.

### Effect of HAMLET on gentamicin uptake and beta lactam binding to pneumococci

**Figure 5:**
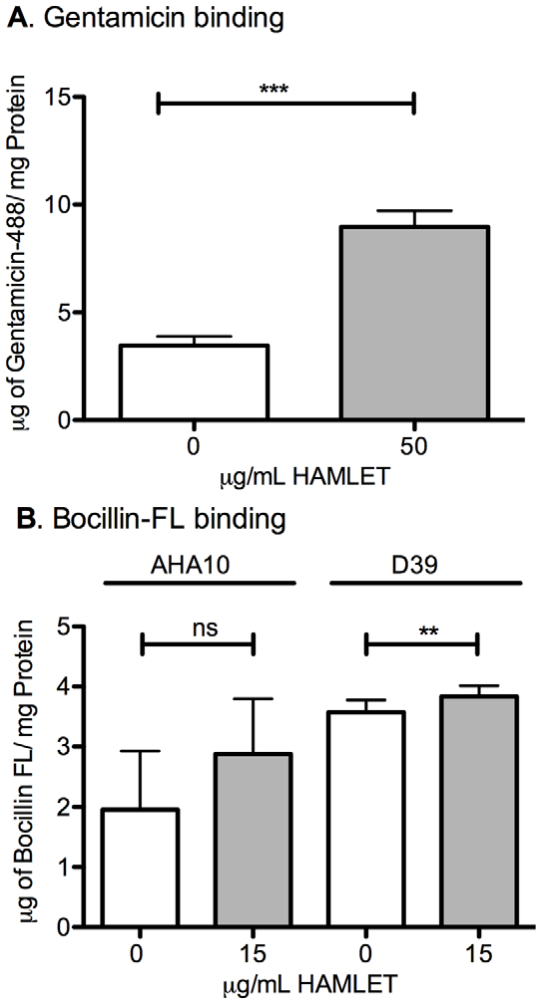
Impact of HAMLET on uptake and binding of gentamicin and Bocillin FL. (A) *S. pneumoniae* D39 were incubated with Alexa Fluor 488-gentamicin in the presence or absence of HAMLET. HAMLET significantly increased the cell-associated level of gentamicin. (B) The penicillin-resistant strain SP670 and the penicillin-sensitive strain D39 were incubated with the fluorescent beta-lactam Bocillin FL in the presence or absence of HAMLET. HAMLET did not increase the cell-associated level of Bocillin FL in either strain. The results are based on three individual experiments with duplicate samples. Statistics was performed using the unpaired Student t-test. Significance was indicated as follows: ** = P<0.01, *** = P<0.001, ns  =  not significant.

In a first attempt to address the mechanism of HAMLET-potentiation of antibiotic activity, we evaluated the effect of sub-lethal HAMLET-treatment on the binding/uptake or cell association of fluorescently labeled gentamicin and the beta-lactam Bocillin FL (a fluorogenic derivative of penicillin V [33], [34]). Bacterial cultures of sensitive D39 pneumococci were incubated with the reporter antibiotics in the presence or absence of subinhibitory concentrations of HAMLET. After allowing the antibiotics to associate with the bacteria, they were washed and lysed by bead-beating, and the fluorescence of the lysate was determined and compared to a standard curve. The addition of 0.75X MIC of HAMLET significantly increased the cell-associated level of gentamicin 2.58-fold (*P*<0.001; **Figure 5A**).

In contrast, sublethal levels of HAMLET had minimal impact on the binding of the beta-lactam Bocillin FL to sensitive D39 pneumococci. The addition of 15 μg/mL HAMLET increased binding of Bocillin FL 1.07-fold compared with the bacteria treated with Bocillin FL alone, which was significantly higher, although not biologically relevant. A more dramatic increase in binding was seen to the penicillin-resistant strain SP670 where the addition of HAMLET increased binding 1.75-fold compared with the Bocillin alone treated culture, however this was not statistically significant (**Figure 5B**).

### HAMLET-induced sensitization of pneumococci to antibiotics requires calcium influx and kinase activation

**Figure 6:**
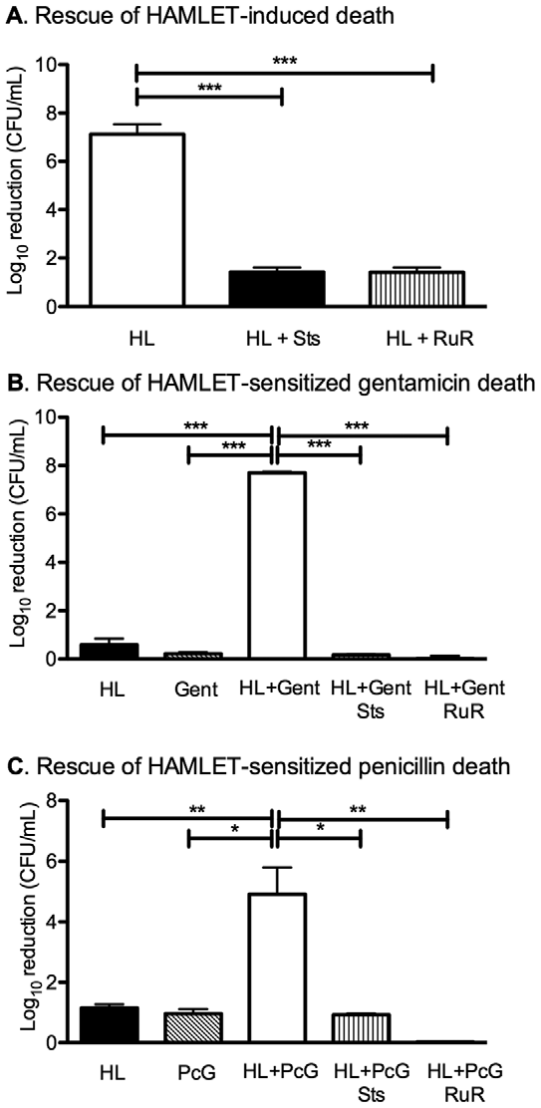
Effect of calcium and kinase inhibitors on HAMLET-induced sensitization of pneumococci to gentamicin. (A) *S. pneumoniae* D39 were treated with a lethal concentration of HAMLET (12X MIC) in the absence of inhibitor (HL) or presence of 20 μM staurosporine (HL + Sts), or 30 µM ruthenium red (HL + RuR) for 1 hour at 37°C. The treated bacteria were diluted and plated on blood agar plates and viable CFU/ml were determined after overnight growth. (B and C) *S. pneumoniae* D39 were treated with 50 µg/mL HAMLET (HL), 50 µg/mL gentamicin (Gent), 20 µg/mL penicillin G (PcG), or a combination of gentamicin and HAMLET or penicillin G and HAMLET in the absence (HL + Gent, HL + PcG) or presence of 20 μM staurosporine (Sts), or 30 µM ruthenium red (RuR) for 1 hour at 37°C. The treated bacteria were diluted and plated on blood agar plates and viable CFU/ml were determined after overnight growth. The graph depicts the log_10_ death induced by each treatment and showed that staurosporine and ruthenium red significantly reduced HAMLET-induced death (A) and also significantly blocked HAMLET's ability to sensitize pneumococci to gentamicin (B) and penicillin G (C). The results are based on three individual experiments with duplicate samples. Statistics was performed using the unpaired Student t-test. Significance was indicated as follows: *** = *P*<0.001.

As it was unlikely that increased antibiotic access was the only mode whereby HAMLET potentiates the bactericidal activity of antibiotics, we further analyzed known HAMLET effector functions. Calcium transport inhibition with ruthenium red, sodium/calcium exchange inhibition with amiloride and dichlorobenzamil (DCB), and kinase inhibition with staurosporine have all been shown to reduce the loss of membrane potential, reduce calcium influx in *S. pneumoniae* in response to HAMLET at lethal concentrations and protect pneumococci from HAMLET-induced death (**Figure 6A**; [35]). To evaluate whether the same activation pathway was involved in HAMLET-induced antibiotic sensitization in pneumococci, when non-lethal concentrations of HAMLET were used, we performed a short time kill assay (1 hour incubation time) using gentamicin and HAMLET or penicillin G and HAMLET-combination treatment, in the presence of staurosporine (20 µM) or Ruthenium Red (30 µM). Both inhibitors completely abolished HAMLET's antibiotic potentiation effect on both gentamicin and penicillin G (**Figure 6B and C**). These results suggest that the same pathway used when HAMLET induces bactericidal activity alone play a critical role for HAMLET's antibiotic potentiation effects.

### HAMLET-antibiotics combination therapy does not result in pneumococcal lysis

**Figure 7:**
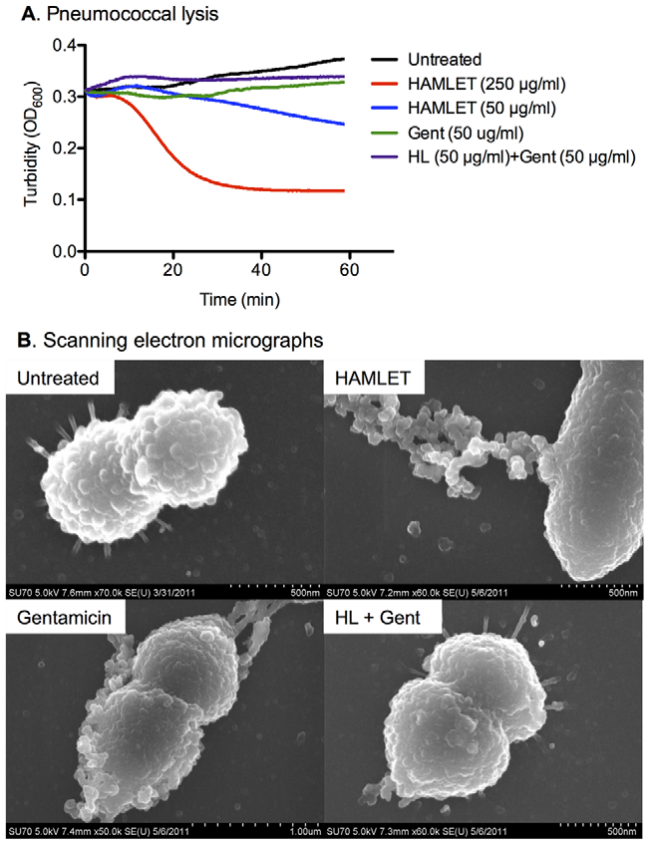
Autolysis during HAMLET-gentamicin combination therapy. (A) Optical density at 600 nm of *S. pneumoniae* D39 (black line) after exposure to a lethal concentration of HAMLET (250 µg/ml; red line), a sublethal concentration of HAMLET (50 µg/ml; blue line), a sublethal concentration of gentamicin (50 µg/mL; green line) or the combination of sublethal concentrations of HAMLET and gentamicin (purple line) that resulted in complete death of the inoculum. The data shows a representative experiment. (B) Representative scanning electron micrographs of untreated *S. pneumoniae* D39, as well as bacteria treated 4 minutes with a lethal concentration of HAMLET (250 µg/mL), one hour with a similarly lethal concentration of gentamicin (500 µg/mL) or one hour with a sublethal concentration of HAMLET (50 µg/mL) or a sublethal concentration of gentamicin (50 µg/mL) in combination (HL + Gent). Note the numerous defects of the pneumococcal cell wall after exposure to HAMLET or gentamicin alone compared with the structurally intact cells after exposure to the combination of the two agents.

Most agents that kill pneumococci, including HAMLET and cell wall-active anti-bacterials activate the major autolysin LytA to induce lysis of the bacteria, which can easily be detected by eye as a clearing of the bacterial suspension upon treatment and measured by a decrease in OD_600 nm_ over time [36], [37]. In comparison, antibiotics acting on bacterial DNA, RNA, or protein synthesis show reduced, though still significant, levels of cell lysis [38], [39]. However, during our combination treatments with HAMLET and gentamicin above we observed no detectable lysis. To quantitate this phenomenon, autolysis of pneumococcal strain D39 was quantified in response to HAMLET alone or a combination treatment with HAMLET and Gentamicin (50 μg/mL each) using optical density. HAMLET alone at a lethal concentration (250 µg/ml) produced a rapid lysis of the inoculum, whereas the combination of sublethal concentrations of HAMLET and gentamicin, that induced equal level or death as the high concentration of HAMLET alone, produced no change in OD_600_ (**Figure 7A**). Even a non-bactericidal concentration of HAMLET (50 µg/ml) alone resulted in a small decrease in optical density at 600 nm (OD_600_) whereas gentamicin at a low concentration failed to lyse the bacteria (**Figure 7A**). Scanning electron microscopy of pneumococci after treatment with either agent alone or in combination confirmed that while HAMLET-induced killing of *S. pneumoniae* results in autolysis of the bacterial cells, combination treatment with HAMLET and another antibiotic did not result in lysis and only intact cells were observed (**Figure 7B**). The lack of lysis from combination treatment may be beneficial for the host in reducing the host inflammation associated with bacterial components after lysis.

### HAMLET combination treatment is active also on species resistant to HAMLET-induced death

During our studies of HAMLET's bactericidal activity we observed that species resistant to HAMLET respond to HAMLET-treatment with depolarization of the bacterial membrane, but that this depolarization did not trigger bacterial death [35]. Depolarization and ion transport is also observed in pneumococci treated with concentrations of HAMLET that not trigger death. We were therefore interested in examining whether the membrane signaling induced in HAMLET-resistant species could also potentiate antibiotic activity. Thus, we investigated the MIC values for two gram-negative respiratory pathogens, *Moraxella catarrhalis* and *Acinetobacter baumanii*, with high levels of inherent resistance to antibiotics.

Both *M. catarrhalis* strain 7169 and BC8 had MICs for HAMLET that exceeded 750 µg/ml and MICs for penicillin that exceeded 50 µg/ml. In the presence of 50 µg/ml HAMLET the MIC for penicillin was reduced over 32-fold to 1.56 µg/ml for strain 7169 and over 4-fold to 12.5 µg/mL for BC8 (**Table 1**). The MIC for gentamicin was 25 µg/ml in both strains. In the presence of 50 µg/ml of HAMLET the reduction in gentamicin MIC in these strains was also significant, but less pronounced (about 4-fold; **Table 1**). Similarly, treatment of *A. baumanii* AB307 and AB979 that had MICs for HAMLET that exceeded 1,000 µg/ml and MICs for penicillin that exceeded 100 µg/ml, the presence of 50 µg/ml of HAMLET reduced the MIC of penicillin 4-fold for both strains (**Table 1**). The MICs for gentamicin was 36 µg/ml and 2.5 µg/ml for strains AB307 and AB979, respectively, which were lowered 8-fold and 2-fold respectively in the presence of 50 µg/ml of HAMLET (**Table 1**). The potentiating effect of HAMLET for all species against gentamicin was abolished in the presence of 30 µM RuR, suggesting that the same mechanism that is activated by HAMLET in pneumococci is activated in other bacterial species.

Combined these results indicate that HAMLET can potentiate the effects of antibiotics in strains that are resistant to HAMLET's bactericidal effect and suggest that the initial depolarization and ion transport events important for both bacterial death and antibiotic potentiation in pneumococci is activated in other organisms.

## Discussion

In this study we have shown that sublethal levels of HAMLET dramatically increase the efficacy of broad spectrum antibiotics, belonging to three different classes, against both pneumococci and HAMLET-resistant species, reducing their MIC and increasing short time killing activity, as well as increasing effects on biofilm growth and nasopharyngeal colonization, with highest efficacy against antibiotic-resistant strains.

Although several concentration combinations were tested for the MIC assays for pneumococci, none met the definition of synergy using an FIC index where synergy is defined as FIC ≤0.5 [40]. This was because MIC assays required 70–75% of the MIC of HAMLET to induce potentiation and reduce the amount of traditional antibiotic required for this species. However, synergy was observed in the short time kill assay, where sublethal levels of HAMLET in combination with gentamicin, penicillin or erythromycin were able to dramatically reduce the amount of antibiotic required to kill pneumococci. These apparently contradictory results are similar to those seen in MIC assays investigating interactions between gentamicin and penicillin or the combination of gentamicin and vancomycin, vancomycin and a quinolone, or combinations of fluoroquinolones and a macrolide with different β-lactams, against *S. pneumoniae*, where MIC assays showed indifference (FIC 0.5–1.0) or limited synergy, but short time kill assays and *in vivo* experimental infections demonstrated synergy [41]–[45]. This underlines the fact that the short time kill assays in vitro correspond more closely to the situation in vivo [42], and that experiments testing antibiotic action against biofilms and in vivo colonization and infection are important to show true efficacy of combination therapy. This is further highlighted by the fact that no FIC value could be determined for the HAMLET-resistant species as an MIC value for HAMLET could not be produced. HAMLET acted as an effective adjuvant in these assays, which the FIC synergy assay cannot measure.

Our studies showed stronger potentiation effects of combination treatment than have been reported previously [41]–[45] and also had the advantage that the time needed to obtain significant synergistic effects in time-kill assays were considerably shorter than those reported by others, with rapid synergism detected as early as 1 hour in the combination of HAMLET and gentamicin and within 4 hours for the combination of HAMLET and erythromycin or penicillin. By reducing the time required for pathogen inactivation, the possibility of establishing further infection is reduced.

This study also represents the first investigation into the effectiveness of combination treatment on *in vitro* biofilms and eradication of pneumococcal colonization *in vivo*, the main pneumococcal niche. Combination of HAMLET and gentamicin, penicillin or erythromycin, significantly enhanced bacterial killing against both antibiotic sensitive and resistant biofilm-embedded organisms in vitro. Our in vivo assays yielded similar results. Addition of HAMLET to gentamicin resulted in a 10-fold reduction in the dose needed to eradicate both lavage- and tissue-associated gentamicin-tolerant pneumococci in a mouse model of established nasopharyngeal colonization and addition of HAMLET to penicillin resulted in a 33-fold reduction in the dose needed to eradicate colonization by the penicillin-resistant strain SP670 that was completely insensitive to penicillin alone.

Current estimates suggest that more than 65% of all human bacterial infections are the result of microbial growth as biofilms [46]. Despite the availability of antimicrobial agents with excellent *in vitro* activity, the treatment of pneumococcal biofilm infections remains problematic. Pneumococcal biofilms have recently been implicated both in infection and colonization where they become 10 to 1000 times more resistant to antibiotics both in vitro and in vivo [27]–[32], [46], [47]. Our results therefore suggests that HAMLET has the potential to restore the effectiveness of β-lactam and macrolide antibiotics against resistant populations, extending their useful life and spectrum, as well as potentially decreasing the therapeutic dose of antibiotic-sensitive species that would result in less pressure for resistance spread in the population.

Although HAMLET has a narrow spectrum of action, showing direct bactericidal effects only against *S. pneumoniae* and *H. influenzae*, membrane effects have been detected in most organisms tested such as *Staphylococcus aureus* and *Escherichia coli* [35]. We therefore tested whether HAMLET could also potentiate the action of antibiotics in HAMLET-resistant bacteria and found it to work equally well or even better than for pneumococci. We focused on the gram-negative respiratory organisms *A. baumanii* and *M. catarrhalis* that both show a high level of antibiotic resistance against beta-lactams and other classes of antibiotics [48]–[51], also being resistant to HAMLET, and showed that the MIC for penicillin and gentamicin were significantly, and dramatically reduced. Thus, HAMLET has the potential to provide a way to increase the usefulness of existing drugs, and extend the lifetime of the current treatment arsenal also in HAMLET-resistant organisms.

To address the mechanism for the dramatic synergy seen between HAMLET and other antibiotics, we first investigated the impact of sublethal levels of HAMLET on antibiotic access to their bacterial targets. Our results indicate that HAMLET significantly facilitated association of gentamicin to the bacteria. However, there was no similar association for beta-lactam binding. Therefore, while increased binding and uptake of antibiotics appears to play some role in the synergistic phenotype seen in combination with HAMLET it does not appear to represent the entire effect. Instead, our study revealed parallels with features of HAMLET-induced death. HAMLET-induced death requires a sodium-dependent influx of calcium that leads to depolarization of the bacterial membrane and activation of a serine/threonine kinase [35]. This activation mechanism results in morphological features similar to apoptotic death in tumor cells [52]. Our results show that reagents that block HAMLET-induced death, such as the calcium transport inhibitor ruthenium red and the kinase inhibitor staurosporine, also abolishes HAMLET's synergic effects with antibiotics. This suggests that specific ion fluxes and kinase activation are required for both HAMLET-induced pneumococcal death and sensitization to antibiotics. How activation of these biochemical events lead to potentiation of various classes of antibiotics is not clear. We have recently shown that the initiation mechanism activated by HAMLET is also activated and required for induction of death by starvation [35], and potentially by antibiotic treatment alone, indicating that an inherent cell death “program” may exist that executes cell death with apoptosis-like morphology. The same is true for other species of bacteria that also show apoptosis-like features when dying from starvation [35]. HAMLET may therefore initiate signaling that lowers the threshold of activation of the inherent cell death program induced by other agents, such as antibiotics. Alternatively, HAMLET may confer bacterial stress that lowers the concentration needed to trigger death with antibiotics in bacteria.

Both HAMLET and antibiotics induce pneumococcal death that culminates in cell lysis and requires the autolysin associated with fratricide [53], although autolysin-negative pneumococci are equally sensitive to these agents as wild type pneumococci [52]. In contrast, an equally lethal combination of HAMLET and gentamicin resulted in a rapid bactericidal effect that was not bacteriolytic. The specific mechanism of this phenotype is unknown, mainly because the activation mechanism of autolysin is unknown, but may result from a down-regulation of the lytic effect from the appropriately balanced inhibition of protein synthesis by gentamicin and activation of HAMLET's conserved apoptotic-like pathway. These interactions result in a final balance where death results in a structurally intact bacterium rather than lysis. HAMLET's ability to prevent bacteriolysis may have numerous positive implications for therapy. When bacteriolysis occurs *in vivo*, cell wall- and membrane-associated lipoteichoic acid, peptidoglycan and pneumolysin are released. Pneumolysin and other cell wall proteins may exert direct cytotoxic effects on eukaryotic cells and act on macrophages to induce the generation and release of damaging reactive oxygen and nitrogen species, cytotoxic cytokines, hydrolases and proteinases in addition to activating the complement cascade [54]–[56]. These sequelae play a prominent role in morbidity and mortality resulting from bacterial infection and therapeutic strategies preventing bacteriolysis in vivo may represent an important avenue for the improvement of microbial therapy [57].

Taken together, these findings may begin to explain the mechanism of the synergy between HAMLET and the antibiotics gentamicin, erythromycin, and penicillin. The results in this system demonstrate that combining a natural component of human milk that has already been used in mice, rats and humans as an anti-cancer therapy without showing any toxic side effects [58]–[60] with traditional antibiotics, and thus targeting the bacteria simultaneously by separate mechanisms, synergistically produces antimicrobial activity that is greater than when each of the active compounds are used individually. This opens up the possibility to use HAMLET or HAMLET's activation pathway to potentiate antibiotic treatment and potentially treat antibiotic-resistant strains of various disease-causing species and extend the use of the current treatment arsenal.

## Materials and Methods

### Reagents

Cell culture reagents, Bocillin FL and the Alexa Fluor 488 labeling kit were from Invitrogen, Carlsbad, CA. Bacterial and cell culture media and reagents were from VWR Inc, Radnor, PA. Chemically defined bacterial growth medium (CDM) was obtained from JRH Biosciences, Lexera, KS. Sheep Blood was purchased from BioLink, Inc, Liverpool, NY. All antibiotics and remaining reagents were purchased from Sigma-Aldrich, St. Louis, MO.

### Production of HAMLET

HAMLET was produced by converting native alpha-lactalbumin in the presence of oleic acid (C18:1) as described [26]. HAMLET was generously provided by Dr. Catharina Svanborg. Lund University, Lund, Sweden.

### Cells and Bacterial Strains

NCI-H292 bronchial carcinoma cells (ATCC CCL-1848) were grown on various surfaces as described [23]. Pneumococcal strains were grown in a synthetic medium (CDM) or in Todd Hewitt medium containing 0.5% yeast extract (THY) as described [61]. The study used the serotype 19F strain EF3030 [62], the serotype 2 strain D39 [63], its unencapsulated derivative AM1000 [64], the D39 derivative lacking PspA through an insertion of an Erythromycin containing resistance cassette (JY53) [65] and a clinical penicillin-resistant pneumococcal serotype 6 strain SP670 [66]. *Acinetobacter baumanii* and *Moraxella catarrhalis* strains were generously provided by Dr. Anthony Campagnari, University at Buffalo, SUNY [67]. *A. baumanii* strains AB307 and AB979 and *M. catarrhalis* strains 7169 and BC8 were cultured in Mueller-Hinton (MH) medium at 37°C with rotary shaking at 225 rpm and stored at −80°C in 50% MH broth and 50% glycerol.

### In vitro susceptibility tests

Minimal inhibitory concentrations (MICs) were determined in 96-well microtiter plates using the microdilution method according to approved standards of the CLSI except that Todd-Hewitt medium supplemented with 0.5% yeast extract, which yields reproducible MIC results was used as the test medium for *S. pneumoniae* [68], [69]. MICs for *A. baumanni,* and *M. catarrhalis* were determined in MH media. Two-fold dilutions of a starting antibiotic concentration was added in triplicate into microtiter plate wells (in 96-well plates), were seeded with a final bacterial concentration of ∼10^5^ colony forming units (CFU)/mL, and was incubated for 18 h in ambient air at 37°C in a Synergy II microplate reader (Biotek, Winooski, VT) where the OD_600_ was recorded every 5 minutes to monitor bacterial growth. The MIC was defined as the lowest concentration of antimicrobial agent solution at which no increase in OD_600_ was detected.

### Short-time kill assays

In late logarithmic growth phase, the bacteria were harvested by centrifugation at 12,000× *g* for 10 minutes and resuspended in phosphate-buffered saline (PBS; 30 mM Na_2_HPO_4_, 10 mM KH_2_PO_4_, 120 mM NaCl, pH 7.4). Appropriate concentrations of the bacteria (around 10^8^ colony forming units per ml) were suspended in PBS and treated with indicated concentrations of HAMLET and/or antibiotics for various times. The effect on bacterial viability was assessed by plating serial dilutions of bacterial sample on tryptic soy agar plates containing 5% sheep blood (viable counts) and determining viable CFUs after overnight growth at 37°C. A bactericidal activity was defined as a reduction of at least 3 log_10_ of the original inoculum.

### Static Biofilm Model

Pneumococci were grown in CDM to mid-logarithmic phase (OD_600_  = 0.5), washed, and resuspended in fresh pre-warmed medium to a density of 2×10^4^ CFUs in 500 µl volume, and suspensions were used to seed sterile round glass coverslips in the bottom of polystyrene 24-well plates with a substratum of confluent H292 epithelial cells as described [27]. Biofilms were cultured at 34°C in 5% CO_2_ for indicated times with change of culture media every 12 hours and used for SEM studies or to assess biomass and antibiotic resistance by viable plate counts.

To test antibiotic sensitivity of the biofilms, pre-formed biofilms were washed with PBS to eliminate planktonic bacteria and were exposed to PBS with indicated concentrations of HAMLET and/or antibiotics for 3 hours at 34°C in 5% CO_2_. Biofilms were then washed in PBS, dispersed by sonication, and collected by pipetting in 100 μL PBS followed by a rinse with 100 μL PBS. Collected cells were then vortexed twice for 20 seconds at high speed and the dispersed biofilm cells were used to determine viable CFUs per ml from diluted samples plated and grown on blood agar. Results are reported as the total number of CFUs per biofilm.

### Ethics statement

This study was carried out in strict accordance with the recommendations in the Guide for the Care and Use of Laboratory Animals of the National Institutes of Health. The protocol was approved by the Institutional Animal Care and Use Committee at the University at Buffalo, Buffalo, NY, USA (Protocol number: MIC36048Y). All bacterial inoculations and treatments were performed under conditions to minimize any potential suffering of the animals.

### HAMLET potentiation of Gentamicin and Penicillin In Vivo

Six-week-old female BALB/cByJ mice from Jackson Laboratories (Bar Harbor, ME, U.S.A.) were maintained in filter-top cages on standard laboratory chow and water ad libitum until use.

Mice were colonized as described previously [61]. In short, 20 µl of a bacterial suspension containing 5×10^6^ CFUs of EF3030 pneumococci in PBS or 1×10^8^ CFUs of SP670 pneumococci in PBS were pipetted into the nares of non-anesthetized mice. After 48 hours, mice were treated with 20 µl gentamicin (0–5,000 µg/mL) or 20 µl penicillin (0–5,000 µg/mL) in the presence or absence of 100 µg (5,000 µg/mL) HAMLET in the nares for 6 hours. Colonization burden was then assessed after euthanizing the animals by enumerating viable bacteria both in a nasopharyngeal lavage obtained by injecting 100 μL PBS in the trachea of mice and collecting it as it flowed out the nares, and from harvested nasopharyngeal tissue after the nasal wash. Nasopharyngeal tissue was dissected out as described [27], [61] by removing the upper skull bone, and harvesting the tissue present in the nasal conchae with forceps. Bacterial load was measured by determining viable plate counts from the nasal lavage or from homogenized tissue.

### Scanning Electron Microscopy

Planktonic bacteria or biofilms grown in vitro (see above) were fixed using 2.5% glutaraldehyde, 0.075% ruthenium red and 0.075 M lysine acetate in 0.1 M sodium cacodylate buffer, pH 7.2 for 1 hr at room temperature. This procedure has been shown to retain carbohydrate structures and improve preservation of biofilm morphology [70]. Samples were washed three times without shaking for 15 min at room temperature in 0.075% ruthenium red in 0.2 M sodium cacodylate buffer and were then dehydrated with a graded series of ethanol (10, 30, 50, 75, 95, and 100%) at room temperature with 15 min used for each step. Samples were exchanged into 100% hexamethyldisilazane, and allowed to air dry before being mounted onto stubs, carbon coated and analyzed using an SU70 Scanning Electron Microscope at an acceleration voltage of 5.0 kV available through the South Campus Instrumentation Center, University at Buffalo, NY.

### Conjugation of Gentamicin with Alexa Fluor 488

Conjugation of gentamicin with Alexa Fluor 488 was performed using the Alexa Fluor 488-conjugation kit (Invitrogen), adapted from the manufacturer's instructions. Alexa Fluor 488 ester was added to a rapidly stirred solution of 0.1 M sodium bicarbonate, pH 8.3 and 10 mg/mL gentamicin and was incubated for 5 hr at 4°C. A gentamicin/Alexa Fluor molar ration of 10∶1 was used to minimize formation of multiply substituted Alexa-Fluor 488-gentamicin conjugates. After conjugation, the conjugated gentamicin was separated from unreacted dye using a provided desalting resin. The concentration of the final product was estimated using the molar extinction coefficient of Alexa Fluor 488. 488-conjugated gentamicin was shown to retain its anti-microbial activity in the conjugated form and was stored at 4°C until used.

### Gentamicin and Bocillin FL Binding

We adapted a previously described method [42] using Alexa Fluor 488-gentamicin, and Bocillin FL as reporter antibiotics to investigate association of these compounds with the pneumococcal cells. In brief, indicated strains were grown in THY medium to an optical density at 600 nm of 0.5. Antibiotics were added at the following concentrations: gentamicin at 50 μg/mL, Bocillin FL at 2 μg/mL, or a combination of gentamicin 50 μg/mL and HAMLET 50 μg/mL, or Bocillin FL 2 μg/mL and HAMLET 15 μg/mL.

After 30 minutes, cultures were centrifuged at 9000× *g* for 4 min, washed four times with PBS and ground with 0.2 µm glass beads for 15 min using a Mini bead beater (Biospec Products Inc). The cultures were then resuspended in a small volume of saline. The Bocillin FL or 488-gentamicin concentration was determined before and after grinding by measuring the amount of fluorescent material in a Synergy II microplate reader (Biotek, Winooski, VT) using excitation and emission wavelengths set at 485 and 530 nm, respectively. For Alexa Fluor 488-gentamicin, linearity was obtained between 2 and 800 μg/mL; (R^2^ = 0.9983) with binding values expressed as a ratio of the sample protein content. For Bocillin FL, linearity was obtained between 0.5 and 100 μg/mL; (R^2^ = 0.9928) with binding expressed as a ratio of the fluorescence of each sample divided with the sample protein content.

### Statistical Analysis

The data in all were analyzed for statistical significance by a two-tailed Student's t-test for paired or unpaired data, as appropriate. A *P-*value <0.05, was considered significant.
